# Celiac Disease and Autoimmune-Associated Conditions

**DOI:** 10.1155/2013/127589

**Published:** 2013-07-24

**Authors:** Eugenia Lauret, Luis Rodrigo

**Affiliations:** Gastroenterology Unit, Central University Hospital of Asturias (HUCA), Celestino Villamil, 33006 Oviedo, Principality of Asturias, Spain

## Abstract

Celiac disease (CD) is frequently accompanied by a variety of extradigestive manifestations, thus making it a systemic disease rather than a disease limited to the gastrointestinal tract. This is primarily explained by the fact that CD belongs to the group of autoimmune diseases. The only one with a known etiology is related to a permanent intolerance to gluten. Remarkable breakthroughs have been achieved in the last decades, due to a greater interest in the diagnosis of atypical and asymptomatic patients, which are more frequent in adults. The known presence of several associated diseases provides guidance in the search of oligosymptomatic cases as well as studies performed in relatives of patients with CD. The causes for the onset and manifestation of associated diseases are diverse; some share a similar genetic base, like type 1 diabetes mellitus (T1D); others share pathogenic mechanisms, and yet, others are of unknown nature. General practitioners and other specialists must remember that CD may debut with extraintestinal manifestations, and associated illnesses may appear both at the time of diagnosis and throughout the evolution of the disease. The implementation of a gluten-free diet (GFD) improves the overall clinical course and influences the evolution of the associated diseases. In some cases, such as iron deficiency anemia, the GFD contributes to its disappearance. In other disorders, like T1D, this allows a better control of the disease. In several other complications and/or associated diseases, an adequate adherence to a GFD may slow down their evolution, especially if implemented during an early stage.

## 1. Introduction

Celiac disease (CD) is a chronic immune-mediated disorder triggered by the ingestion of gluten that appears in genetically predisposed patients The clinical spectrum of CD is wide and includes classic presentation of malabsorption with diarrhea, nonclassical extraintestinal features, subclinical or asymptomatic forms, and potential disease characterized by positive serology with a normal intestinal mucosa on biopsy [1, 2]. Moreover, a significantly increased prevalence of other autoimmune diseases (AD) has been reported in individuals with CD and their first-degree relatives as compared to controls [3–7], with an estimated burden of AD in CD cases up to 15% [7]. In celiac patients, an early diagnosis in life and having a family history of autoimmunity are risk factors for developing other AD, while the gluten-free diet (GFD) has a protective effect [8]. By contrast, in relatives of CD cases, the prevalence of AD rises with the age [5]. Conversely, a significantly increased prevalence of CD has been documented in individuals with other AD [9, 10]. It has been suggested that these associations among CD and other AD may be explained by the sharing of a common pathogenic basis involving genetic susceptibility, similar environmental triggers, and the loss of intestinal barrier secondary to dysfunction of intercellular tight junctions with increased intestinal permeability, and possibly by other undiscovered mechanisms [7, 11–16]. In this review, we present a detailed description of the main AD associated with CD (Table 1). 

## 2. Immunogenetics of Celiac Disease

The name of gluten is applied to a collective set of proteins for the storage that are found in grains of wheat, barley, and rye [17]. 

Typically, gluten proteins are rich in glutamine and proline residues. Their high proline content makes them resistant to gastrointestinal digestion. In wheat, gluten proteins are divided into gliadins and glutenins, whereas the gluten proteins of barley and rye are termed hordeins and secalins, respectively. Patients with CD raise CD4+ T cell response against several distinct gluten peptides and these peptides are recognized in the context of CD-associated HLA-DQ molecules [18]. In addition, patients make antibodies specific for gluten proteins.

Autoantibodies in CD were initially detected as reticulin-specific antibodies by staining of rat tissue [19]. Subsequently, IgA endomysium-specific antibodies (EMAs), detected by staining of monkey oesophagus or human umbilical cord, were described [20]. The main antigen recognized by reticulin-specific antibodies and EMAs was identified as the enzyme transglutaminase 2 (TG2) in 1997, by Dieterich et al. [21]. 

TG2-specific antibodies are found in serum as IgA and IgG isotypes. Assaying for TG2-specific IgA is most commonly used in clinical practice, as this test has the highest disease specificity and sensitivity. In fact, the disease specificity and sensitivity for this test are higher than for any other auto-antigen-disease association. Previously, the detection of histological changes in small intestinal biopsy samples, such as blunting of intestinal villi and infiltration of inflammatory cells, was considered mandatory for the diagnosis of CD. 

TG2 antibodies are detected in 0.3–2% of individuals in Europe and in the US, and comparable prevalence rates are observed in a growing number of countries in which gluten is an important component of the diet [22, 23]. Positive serology is more particularly frequent in first-degree relatives, a finding consistent with the importance of genetic predisposing factors, and in patients with AD, notably with type 1 diabetes (T1D) and thyroiditis. In individuals with positive serology, the diagnosis of CD is confirmed by duodenal biopsies that demonstrate typical small intestinal lesions combining villous flattening, crypt hiperplasia and increased numbers of intra-epithelial lymphocytes (IELs).

Several studies highlighting a strong association between CD and the HLA complex and it was established in the late 1980s, with HLA-class II associations for CD and T1D. The majority of CD patients express the *HLA-DQ2.5* heterodimer encoded by the HLA-DQA1*05 (alpha-chain) and HLA-DQB1*02 (beta-chain) alleles. Both are carried either “in cis” on the *DR3-DQ2* haplotype, or “in trans” in individuals who are *DR5-DQ7* and *DR7-DQ2* heterozygous. *HLA-DQ8* that is encoded by the *DR4-DQ8* haplotype, confers a lesser risk of CD. In individuals who are *DR3-DQ2* and *DR4-DQ8* heterozygous, transdimers *DQ8.5* can form and confer a high susceptibility to T1D. Therefore, genetic susceptibility to CD is determined mainly by the HLA-DQ locus [24]. The strongest association is observed with *HLA-DQ2.5*; thus more than 90% of celiac patients possess one or two copies of *HLA-DQ2.5* [25]. 

Gut lesions of most patients with CD completely normalize when gluten is excluded from the diet, and they reappear when the patients eat gluten again. Both the presence of autoantibodies specific for TG2 and the increased number of intraepithelial cytotoxic T lymphocytes (IE-CTLs) are gluten dependent, as they change after gluten elimination or challenge [26]. Patients with CD, but not healthy controls, have *HLA-DQ2* or *HLA-DQ8* restricted CD4+ T cells in the gut mucosa that are reactive to gluten [27]. Given the strong HLA association, CD4+ T cells are probably key players in the development of the disease.

Another key aspect of CD is the up-regulation of certain interleukins mainly IL-15 type, and in combination with nonclassical MHC class I molecules in the epithelium. It is probable that the CD4+ T cell response has a role, but it is not sufficient alone to induce these alterations in the intestinal epithelium. Together, these observations are in line with the concept that a strong adaptive immune response is typically paired with an innate component that facilitates the adaptive response [28]. 

IE-CTLs can destroy the intestinal epithelial cells in CD on the basis of the presence of stress signals, rather than because they are specific for an epithelial cell antigen. Only cells that up-regulate IL-15 and nonclassical MHC class I molecules will be targeted by IE-CTLs. The receptor-ligand interactions involve noncognate recognition of low affinity self or microbial peptides by the T-cell receptor (TCR) and recognition of inducible nonclassical MHC class I molecules by NK cell receptors. Thus, the presence of CD8+ T cells with lymphokine-activated killer activity and/or a very low activation threshold in the tissue targeted by an autoimmune process could point to an exogenous factor, which probably would be the gluten.

In summary, gluten has a dual effect upon the small bowel mucosa. By one side, small toxic peptides as the 19-mer, induce an innate unspecific immunologic response, characterized by the presence of IL-15 produced by the enterocytes. This interleukin promotes the liberation of the transcription factor NF-*κ*B by the adjacent cells that increase the IL-15 production and the induction of nitric oxide synthase (iNOS), promoting oxidative stress and helping to maintain the innate response. Different molecules such as MICA and/or HLA-E are increased at the enterocytes and induce their apoptosis. Finally, IL-15 opens the *tight*-*junctions* between the epithelial cells. 

**Figure 1:**
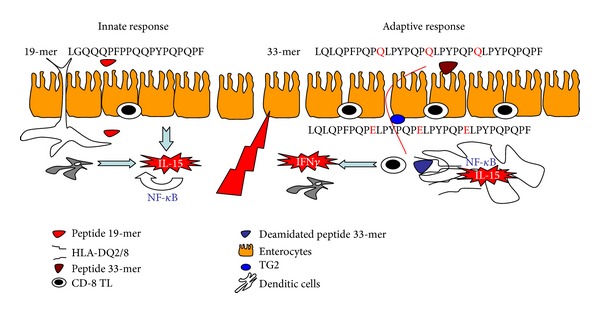
Gluten has a dual effect on the small intestine mucosa. *Innate response* (left). Toxic peptides, such as the 19-mer, induce an unspecific immune response characterized by the presence of IL-15 produced by the enterocytes, that in turn activates the NF-*κ*B in the adjacent cells, which enhances the IL-15 production and iNOS induction and feedback of the innate response. Molecule expression as MICA and/or HLA-E is increased in the enterocytes and IL-15-triggered apoptosis on these cells to induce expression of NKG2D and NKG2C molecules (ligands MICA and HLA-E, resp.) in intraepithelial lymphocytes. Finally, IL-15 can weaken the bonds tight-junctions between the enterocytes. *Adaptive response* (right): is facilitated by increased intestinal permeability allowing passage of immunogenic peptides such as the 33-mer to the lamina propria, which are deamidated by the enzyme tissue transglutaminase (TG2). Furthermore, IL-15 activates dendritic cells, which increases the surface expression of costimulatory molecules, necessary for effective antigen presentation by HLA-DQ2-restricted/DQ8, to T lymphocytes. These lymphocytes trigger a Th1 response, with a predominance of IFN and the absence of IL-10, and the release by stromal cells, growth factors, and keratinocytic metalloprotease of Th1 cytokine profile is responsible for the injury, characterized by intraepithelial lymphocytosis, crypt hyperplasia, and villous flattening, but can also attract new proinflammatory cells in the lamina propria. (Adapted with permission from Dr. E. Arranz).

The adaptive response is performed by the increased permeability which permits that immunologic peptides such as 33-mer arrive to the lamina propria where they are deamidated by TG2. IL-15 activates to dendritic cells for presenting the deamidated gluten peptides by the HLA-DQ2/8 molecules to the CD8+ T lymphocytes. These facts start a Th1 response, liberating IFN*γ* and other metalloproteases. This profile of cytokines is the responsible of the epithelial lesion, characterized by the presence of IELs, crypts hyperplasia, villous atrophy, and chronic inflammatory infiltrate at the lamina propria (Figure 1).

In recent years, the role of prolactin (PRL) as modulator of the immune response has been elucidated. Hyperprolactinemia has been found in the active phase of both nonorgan-specific and organ-specific AD. There is a correlation between PRL level and the number of CD4+ T and B lymphocytes, and this hormone stimulates the release of cytokines and enhances the autoantibody production. The relationship between prolactin and AD could be partially explained because the human prolactin gene is located in short arm of the chromosome 6 close to HLA region [29, 30]. In CD, it was shown that hyperprolactinemia is present in patients in active phase under gluten-containing diet but not in those with nonactive CD following a GFD. Likewise, it was also found a good correlation between serum PRL level with the degree of mucosal atrophy and between the serum concentrations of this hormone and EMAs. For this reason, it has been suggested that PRL level may serve as a potential marker for CD activity [31, 32]. 

As mentioned, CD has a strong HLA-DQ association, and this genetic background play a key role in the predisposition to the disease development with an estimated risk effect of 40%. On the other hand, an unknown number of non-HLA genes contribute to the other 60% of genetic susceptibility. Of the later, variants in the genes cytotoxic T lymphocyte antigen 4 (*CTLA*-*4*) and myosin 1XB (MYO9B) have been associated with CD, but widespread replication of these findings in large cohorts from different populations remains limited. Additionally, in the past few years, two genome-wide association studies (GWAS) and one fine-mapping project have identified up to 57 non-HLA single-nucleotide polymorphisms (SNPs) that contribute to CD susceptibility. However, taken together these genes still only explain up to 10% of the CD heritability, while many others remain undiscovered [33–36]. 

The relationship between a variant at exon-3 (rs1143679) of Integrin-*α*-M (ITGAM) and some AD including CD has also been examined. This gene encodes the *α*-chain of a heterodimeric integral membrane protein, a cell surface receptor that plays an important role in the adherence of neutrophils and monocytes to stimulated endothelium, and also in the phagocytosis of complement coated particles. No association was observed between this genetic variant and CD [37]. 

Despite their phenotypic differences, recent genetic studies have shown shared loci between CD and other AD [35, 38]. This common genetic background in conjunction with environmental factors may partly explain the concurrent development of several immune-mediated disorders in celiac patients and their relatives. So, an overlap of HLA haplotypes between CD and other AD has been described [39, 40]. Similarly, it is estimated that of 39 known CD-associated non-HLA loci, approximately 64% are shared with at least one other AD, most of which encodes several genes involved in inflammatory and immune responses [39]. Some of them may act as regulators of proliferation and activity of T lymphocytes (*CTLA-4*, *ICOSLG*, and *IL18RAP*). Other genes have been implicated in nuclear factor-kappa B activity (*REL, UBE2LE*) and signalling processes *(SOCS1, SH2B3) *or in more than one function such as T lymphocyte and cytokine activities (*IL2, IL21, ILI2A*, and *IL23R*) or T lymphocyte and signalling processes (*PTPN2*) [35, 38, 39]. 

## 3. Autoimmune Disorders in Celiac Disease

### 3.1. Associated Autoimmune Liver Disease

A variety of hepatobiliary disorders have been described in CD. Liver changes were first recognized by Hagander et al. [41], and later, these findings were confirmed in several studies in which abnormal liver enzyme tests were detected in over 20% of cases [41–44]. Conversely, in patients with unexplained elevations of liver enzyme levels, it was estimated that up to 10% of cases such abnormalities were due to CD [43–46]. 

Mild liver abnormalities are common in patients with CD and usually resolve with a GFD [42, 43]. In others, clinically significant liver disease is detected, and these cases are not amenable to diet treatment alone. Furthermore, several studies have demonstrated consistent associations between autoimmune liver diseases and CD, such as primary biliary cirrhosis (PBC), autoimmune hepatitis (AIH), and primary sclerosing cholangitis (PSC) [47]. The histologic features in these patients with CD-associated autoimmune liver diseases were typical of their respective entities [48].

#### 3.1.1. Primary Biliary Cirrhosis

In 1978, Logan et al. described the first cases of PBC in celiac patients [49], and later, this association has been well established in subsequent studies with a prevalence of CD in patients with PBC in around 3–7% [50–52]. Conversely, the frequency of diagnosed PBC in patients with CD is around 3% [51, 52], with a 3-fold to 20-fold increased risk of PBC compared to the general population [47, 53, 54]. 

Both CD and PBC share several features, including a higher prevalence in females, autoimmune comorbidities, and specific autoantibodies. The breakdown of gut-liver axis equilibrium has been implicated as a mechanism underlying this association. In CD, immunologically active molecules generated from the cross-linking between tissue transglutaminase and food/bacterial antigens reach the liver through the portal circulation owing to the increased intestinal permeability. A molecular mimicry between bacterial antigens and the pyruvate dehydrogenase E2 component, recognized by antimitochondrial autoantibodies, may have a role in PBC pathogenesis. An aberrant intestinal T lymphocyte homing to the liver may contribute to trigger immune hepatic damage [55]. 

#### 3.1.2. Autoimmune Hepatitis

A potential association between CD and AIH has also been reported in several publications, with a prevalence of CD in patients with AIH of 3% to 6% [44, 56, 57] and an estimated relative risk in children with AIH of 6.63 (95% CI, 3.86–11.40). However, the reported prevalence of AIH in celiac patients is less than 2% [44].

CD and AIH share selected combinations of genes coding for class II HLA, which could explain their coexistence. Increased intestinal permeability and circulation of antitissue transglutaminase (tTG) have also been considered as further potential causes of liver damage in CD patients. These antibodies in the liver and in other extraintestinal tissues could modify other external- or self-antigens and generate different neoantigens, which are responsible for liver injury in patients with CD [58].

Although the treatment of AIH is based in the use of steroids and/or other immunosuppressive drugs, it has been seen that patients with AIH and CD achieve treatment-free sustained remission in a significantly higher proportion than patients with AIH without CD, suggesting a possible adjuvant effect of GFD [59, 60]. 

#### 3.1.3. Primary Sclerosing Cholangitis

The association between CD and PSC was initially reported in 1988 [61], and later other publications have described this same relationship, with a prevalence of CD in patients with PSC of up to 3% [62, 63]. However, in several cases, the diagnosis of PSC was made in patients with inflammatory bowel disease, so it is not possible to rule out a confounding factor. Nevertheless, Ludvigsson et al. in a large study utilizing the data of Swedish national register, found a 4-fold increased risk of PSC in celiac patients, and both diseases remained significantly associated after exclusion of individuals with inflammatory bowel disease [47]. 

GFD was reported to cause an improvement in hepatic histological characteristics and cholestasis only in a small number of cases, making difficult to confirm whether the diet may slow the progression of this autoimmune liver disorder [64, 65].

#### 3.1.4. Others

*Nonalcoholic Fatty Liver Disease*

Non-alcoholic fatty liver disease (NAFLD) is a very common disorder (up to 25%) in the general population, and its occurrence in patients with CD is likely to be a coincidence rather than a true relationship between both diseases. Some studies have found a prevalence of CD in patients with NAFLD by around 3% [66, 67], and conversely, CD was associated with 6-fold increased risk of fatty liver [47]. GFD helps normalize the liver blood test abnormalities [66], but the effect on the reversal of the histological damage is not clear [48]. 

Recently, it has been suggested that NAFLD is associated with increased gut permeability caused by disruption of intercellular tight junctions in the intestine, and this fact may play an important role in the pathogenesis of hepatic fat deposition [68]. Similarly, as the loss of intestinal barrier with increased intestinal permeability induced by gliadin has also been described as involved in the pathogenesis of CD [15], it is possible that both diseases can relate more than mere coincidence since they seem to share a similar pathogenic mechanism. 

*Wilson's Disease*

In a recent study, in which serological markers for CD were tested in patients with various liver diseases with subsequent confirmation of positive cases by intestinal biopsy, it was found a high prevalence of CD in patients with Wilson's disease [69]. Although this association is considered rare and has not been fully analyzed, abnormalities in copper metabolism in CD with impaired copper uptake from the gut and levels in urine higher than in healthy controls have been reported [70]. 

*Budd Chiari Syndrome*

There are also reported cases of hepatic vein obstruction in celiac patients, particularly from North Africa or Southern Europe [71, 72] and isolation in other locations [73, 74]. This disorder has been related to deficiencies in protein C and antithrombin III, malabsorption of vitamin K, decreased synthesis of coagulation factors dependent on this vitamin such as protein C or protein S, and even with dietary or environmental agents. Recently, it has been suggested that the increased incidence of serum antiphosphatidylserine/prothrombin IgG antibodies and higher rates of activity for antiphosphatidylserine/prothrombin IgM and prothrombin IgG autoantibodies observed in celiac patients may be involved in the hypercoagulability predisposition of CD [75].

### 3.2. Associated Autoimmune Endocrine Diseases

#### 3.2.1. Type 1 Diabetes

The association between CD and autoimmune insulin-dependent diabetes mellitus is one of the most intensely studied. The diagnosis of the two diseases is often simultaneous or CD subsequent to diabetes [76, 77]. The prevalence of CD among patients with T1D has been estimated in approximately 4% (range from 2% to 11%) [22, 76–81], and this risk is highest with diabetes onset in childhood (age < 4 years) but also with the longer diabetes duration [77, 82, 83]. Conversely, it has been described that CD is also associated with an increased risk of subsequent T1D before age 20 years (Hazard ratio (HR) 2.4; 95% CI, 1.9–3.0) [84]. 

Testing for CD should be offered to children with T1D, due to high prevalence in this at risk group with potential consequences of delayed diagnosis [85]. Similarly, there is disclosed a second peak incidence in adult T1D patients around 45 years of age, which emphasizes that screening for CD is also required in adult diabetic patients [86]. 

CD and T1D share HLA risk genotypes. Approximately 90% of individuals with T1D have either *DQ2* or *DQ8*, compared to 40% of the general population [87]. Homozygosity for *DR3-DQ2* in the population with T1D carries a 33% risk for the presence of tTG autoantibodies, and conversely, less than 2% of patients lacking *DQ2* or *DQ8* have CD-related autoantibodies [88, 89].

In patients with both disorders, GFD prevents a grow failure in children and leads to a better metabolic control of diabetes, although with a slight increase in insulin dose due to correction on the intestinal malabsorption and a higher glycemic index of gluten-free products [87, 90, 91]. Furthermore, GFD has a protective effect on the development of vascular complications in T1D patients [92, 93]. Moreover, T1D and CD have a negative effect on bone metabolism in relation with duration and/or poor control of diabetes, nutritional mechanisms/no compliance to GFD, and immunoregulatory imbalance. In fact, in patients affected by both disorders, osteopenia occurs more frequently in patients with poor compliance to GFD, so that it and the optimization of glycemic control play an important role in preventing the osteopenic status caused by the clustering of these two chronic diseases [94, 95]. 

#### 3.2.2. Thyroid Diseases

CD has been found at an increased rate in patients with autoimmune thyroid disease (Grave's disease and Hashimoto's thyroiditis), with a prevalence ranging from 2% to 7% [96–99]. This same observation has been made in patients with CD, in whom serological signs of autoimmune thyroid disease were found up to 26%, occurrence of thyroid dysfunction was detected in up to 10% of cases, and risk of thyroid disease was estimated 3-fold higher as compared to controls [3, 98, 100–102]. 

It has been described that celiac individuals who are following a GFD may still develop autoimmune thyroid impairment, suggesting that gluten withdrawal does not protect them [100, 102–104]. By contrast, the decrease of the thyroid antibodies after 2 or 3 years [105] or the normalization of thyroid function after 1 year of GFD has been reported in other studies [106]. These different results may depend on longer duration of GFD in treated patients with CD [107].

Increased prevalence of CD, autoimmune thyroid disorders, and T1D has been widely reported [22]. Such associations may lead to adverse effects on the growth, metabolism, and fertility, so early detection is necessary to prevent secondary complications to these disorders. 

The coexistence of CD and autoimmune thyroid disease has been explained by several mechanisms such as common genetic predisposition and the association of both diseases with the gene encoding cytotoxic T-lymphocyte-associated antigen-4, a gene conferring susceptibility to thyroid autoimmunity. In addition, it has also been demonstrated that tTG-2 IgA antibodies react with thyroid tissue, and this binding could contribute to the development of thyroid disease in CD [98, 108].

#### 3.2.3. Addison's Disease

Patients with Addison's disease are considered a group at-risk for CD [109–114]. Several studies had reported a high prevalence of CD among patients with this endocrine disease, ranging from 5% to 12% [111–114]. Conversely, this association was also confirmed, showing an increased risk of developing Addison's disease among celiac patients (HR 11.4; 95% CI, 4.4–29.6) [115], and it has been described that GFD does not modify the natural history of Addison's disease [114].

### 3.3. Associated Autoimmune Dermatological Diseases

#### 3.3.1. Dermatitis Herpetiformis

Dermatitis herpetiformis (DH) is an inflammatory cutaneous disease, with typical histopathological and immunopathological findings, clinically characterized by intensely pruritic polymorphic lesions with a chronic-relapsing course, first described by Duhring in 1884 [116]. In 1966, Marks et al. [117] reported the presence of small bowel changes in DH patients, and later on, these gastrointestinal abnormalities, described in affected patients, were found to be the same as in those with CD [118]. It is now currently considered to be the more common and specific cutaneous manifestation of CD. Its presence is characteristic of this disease, and by consequence, its best treatment is a strict lifelong GFD, for achieving and maintaining a permanent control. It appears in around 25% patients with CD, at any age of life, mainly in adults. It can be considered as the “visiting card” of celiac patients, because its finding appears only in CD individuals.

**Figure 2:**
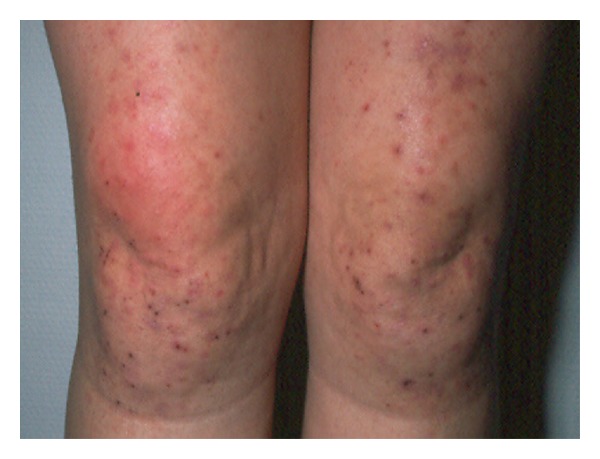
Custry DH lesions in both knees.

**Figure 3:**
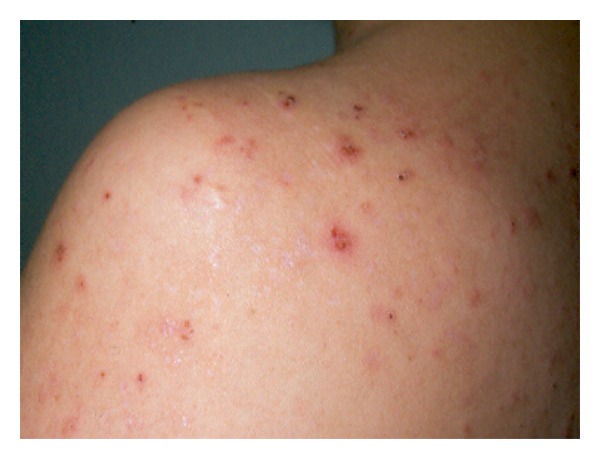
Several evolutive DH lesions in left shoulder and back.

Primary DH lesions are characterized by the presence of grouped erythematous papules, urticarial plaques, surmounted by vesicles, or also blisters, which may be often replaced by erosions and excoriations, because of the intense itching, characteristically associated with this condition. Chronic pruritus and excoriations might lead to its lichenification (Figures 2 and 3). Furthermore, a postinflammatory hyperpigmentation may occur when the lesion resolves [119–121].

The symmetrical distributions of the herpetiform lesions on the extensor surfaces of the elbows (90%), knees (30%), shoulders, middle line of the back, buttocks, and sacral region are the main affected localizations that are the site of constant minor trauma and is also a typical feature of the disease [122]. Anyway, scalp, nuchal area, face, and groins may be also involved. No clinical differences were described between darker and white-skinned individuals, although DH remains primarily predominant in Caucasian population, being rarer in Asian populations, including the Japanese [123]. 

Most of the patients suffer not only of itching, but also tickle or burning sensation, even before the onset of the skin lesions. An uncommon skin manifestation of DH is represented by purpuric lesions, occasionally found on palmo-plantar surfaces of children, but rarely reported in adults. Sometimes, petechial lesions on the fingertips may be the only symptom of DH [124].

However, atypical clinical presentation of DH includes also the presence of palmo-plantar keratosis, wheals of chronic urticarial, and other lesions mimicking prurigo pigmentosa [125, 126].

The classic histopathological features of DH seen on light microscopy include a subepithelial cleft with neutrophils, that are considered the most likely responsible for the dermoepidermal separation and a few eosinophils at the tips of dermal papillae that are accompanied by a perivascular-mixed inflammatory infiltrate. 

The pathophysiology of DH is complex and involves genetic factors such as HLA predisposition (mainly HLA-II, as *DQ2*, and *DQ8*), environment trigger (gluten), and dysregulation of the immune system, in predisposed individuals, as occurs in CD patients without skin affectation [127].

Actually direct immunofluorescence of uninvolved skin biopsies collected in the perilesional site is considered to be the “diagnostic gold standard” for DH [128]. This is because in this location a greater IgA deposition than in nonlesional or lesional skin is found. Two different patterns are found (1) granular deposits in the dermal papillae and (2) Granular deposits along the basement membrane. In both cases, deposits are thought to be polyclonal but are mainly composed of IgA. The two patterns may also be present in combination, resulting in granular IgA deposition along the basement membrane with accentuation at the tips of dermal papillae [129].

Serologic testing is useful adjunct to tissue-based studies. The patients with DH have positivity for the gluten-induced tTG-2 and tTG-3 autoantibodies, also called tissue-TG (tTG) and epidermal (eTG) respectively, together with the deamidated synthetic gliadin-derived peptides (anti-DGP), more sensitive in children [130].

A potential explanation of the appearance of DH lesions is related to the presence of an active chronic small bowel mucosal inflammation, as a result of a persistent gluten challenge, with a local immune response and the production of mucosal IgA. A part of the circulating IgA (anti-tTG3) binds to the skin. Consequently, the gastrointestinal immune response results in increased levels of circulating cytokines, which may attract neutrophils, as well as induce an active Th-2 response in the endothelial cells. Ultraviolet beams (UVB) and minor repeated microtrauma to the skin increase the local cytokines production, leading to the egress of neutrophils, deposition of IgA at dermis and thus to the development of the typical skin DH lesions [131].

Associated AD are more common among DH patients. Family screening for gluten sensitivity is also strongly suggested. Untreated patients should be regularly monitored for malabsorption and lymphomas.

#### 3.3.2. Others

Some cases of alopecia areata associated with CD have been reported [132–134]. The administration of a GFD to these patients has variable results, and not in all cases, the recovery of hair growth with the diet [133] has been described.

The relationship between vitiligo and CD is controversial. Although some cases of both diseases have been described, this association remains unclear [135, 136]. 

Dermatomyositis is a disease characterized by erythematous and edematous changes in the skin and muscle weakness. Some reports have suggested an association between this condition and CD [135, 137, 138] with response to GFD [138].

### 3.4. Associated Autoimmune Neurological Diseases

The first study of patients with CD (confirmed by biopsy) and a neurological deficit was published by Cooke and Smith in 1966 [139]. Since then, a broad spectrum of neurological manifestations has been related to CD, with a prevalence of approximately 10 to 12% [140]. Gluten ataxia (GA) and peripheral neuropathy are the more common related disorders, and these neurological manifestations can present even in the absence of an enteropathy. 

GA can be defined as sporadic ataxia triggered by the ingestion of gluten, with positive serum antigliadin antibodies with or without enteropathy on duodenal biopsy [14]. This disease usually presents with pure cerebellar ataxia or, rarely in combination with myoclonus, palatal tremor or opsoclonus myoclonus. In a study by Hadjivassiliou et al., these authors reported that GA accounted for 36% of cases of idiopathic sporadic ataxia; 72% of these patients had *HLA-DQ2*, but only 24% of them had gluten-sensitive enteropathy, and only 13% reported gastrointestinal symptoms [141]. 

Peripheral neuropathy related to CD is a symmetrical sensorimotor axonal neuropathy and usually presents with burning, tingling, and numbness in hands and feet, with distal sensory loss [142]. It has been described that gluten sensitivity may be the etiology of 34% of cases with idiopathic neuropathy, with a prevalence of biopsy-verified CD at least 9% and presence of HLA types associated with CD in 80% of patients [143]. In a large study with patients from the Swedish national register, the association between CD and later polyneuropathy was confirmed (HR 3.4; 95% CI, 2.3–5.1), and conversely, prior polyneuropathy was a risk factor for subsequent CD (OR 5.4; 95% CI, 3.6–8.2) [144].

One of the mechanisms involved in the GA pathogenesis may be the antibody cross-reactivity between antigenic epitopes on Purkinje cells and gluten proteins. Additionally, while the development of anti-tTG-2 IgA is linked with CD, an anti-tTG-6 IgG and IgA response is prevalent in GA, independent of intestinal involvement. It has been shown that in those patients with ataxia and enteropathy, separate antibody populations react with the two different transglutaminase isozymes. Postmortem analysis of brain tissue showed cerebellar IgA deposits that contained TG-6 [145]. So, these antibodies are gluten dependent and appear to be a sensitive and specific marker of GA, and it has been seen that their titers diminish or disappear with GFD [146, 147]. 

Antibodies to gangliosides and Purkinje cells have been reported in celiac patients with neuropathy and ataxia [148, 149], but the pathogenic role of this finding has not been clearly demonstrated [150, 151].

Early diagnosis and treatment with a GFD can improve both neurological manifestations, although this effect has not been seen in all cases [152, 153]. The response to a diet may depend on the duration of the disorder prior to diagnosis of gluten sensitivity. Loss of Purkinje cells in cerebellum as result of prolonged gluten exposure is irreversible. By contrast, an early treatment is most likely to reverse or stabilize the neurological disease [1]. 

Other neurological disorders such as demyelinating diseases have also been described to be associated with CD. The first mention of a possible relationship between CD and multiple sclerosis is recorded in 1965 [154]. In a Spanish study, the estimated prevalence of CD in patients with multiple sclerosis was 11% [155], although this association was not confirmed in other series [156]. Only in a small number of sporadic cases, the combination CD and neuromyelitis optica was found [157, 158]. GFD is recommended for both disorders, although the long-term neurological response with the diet is not clear [155, 159]. 

### 3.5. Associated Rheumatological Disorders and Connective Tissue Diseases

#### 3.5.1. Sjogren's Syndrome

Sjogren's syndrome (SS) is an autoimmune disease characterized by lymphocytic infiltration and malfunction of exocrine glands that presents with sicca symptoms of mucosa surfaces. Systemic manifestations results from cutaneous, respiratory, renal, hepatic, neurologic, and vascular involvement, with a high risk for progression to lymphoma [160].

Findings of several studies had shown a significant association between SS and CD, confirmed by small bowel disease, with a prevalence rate of CD among patients with SS in the range of 4.5% to 15% [161, 162]. Successful treatment of CD did not relieve sicca symptoms or signs; thus, the two diseases must be treated independently [163].

#### 3.5.2. Systemic Lupus Erythematosus

Systemic lupus erythematosus (SLE) is a multisystem disorder with manifestations including rash, arthritis, cytopenia, and renal disease [164]. 

Some case reports have suggested the association between CD and SLE [165–168]. In a recent study, the aim was to investigate the risk of this disease in a nationwide cohort of patients with biopsy-verified CD compared to controls matched from the general population. The conclusion was that celiac patients were at 3-fold increased risk of developing SLE, but absolute risk was low [169]. This is striking because both disorders share the human leukocyte *HLA-B8* and *HLA-DR3* histocompatibility antigens, and a variety of antibodies including the detection of tTG IgA, antinuclear, and antidouble-stranded DNA antibodies. Furthermore, it has been shown that SLE may develop later in the clinical course of de CD, even after a small bowel biopsy response to a GFD [168].

#### 3.5.3. Juvenile Idiopathic Arthritis and Rheumatoid Arthritis

The term juvenile idiopathic arthritis (JIA) describes a clinically heterogeneous group of autoimmune arthritides characterized by chronic synovitis, which begin before 16 years of age and may be accompanied by other extraarticular manifestations as fever, rash, pericarditis, and uveitis [170]. 

Other associated ADs, such as CD, have been previously described in patients with JIA, with a prevalence rate of 2.5% to 7% [9, 171, 172]. Moreover, a significantly increased cases of JIA in first-grade relatives of celiac patients have been found [7]. 

Conversely, the relationship between CD and rheumatoid arthritis (RA) has not been demonstrated. In the majority of studies, RA was reported as an associated diagnosis in patients with CD in a lower percentage than controls [7, 173, 174].

### 3.6. Miscellaneous

#### 3.6.1. Cardiological Diseases

Dilated cardiomyopathy (DCM) is defined by the presence of left ventricular dilatation and left ventricular systolic dysfunction in the absence of abnormal loading conditions (hypertension, valvular disease) or coronary artery disease sufficient to cause global systolic impairment. Familial and acquired forms have been described, and the latter is related with nutritional deficiencies, endocrine disorders, cardiotoxic drugs, excessive alcohol intake, pregnancy, or infections. Some observations also suggest that immunologic factors might be involved in the pathophysiology of DCM [175]. 

An increased prevalence of CD (up to 5.7%) has been recently recognized in patients with idiopathic DCM [176–179]. Conversely, in a recent nationwide study, it was found that patients with CD had a moderately but not statistically significantly increased risk of idiopathic DCM (HR 1.73; 95% CI, 1.00–3.00) [180]. In these patients, a GFD may have a beneficial effect with improvement of echocardiographic parameters as well as in cardiological features and quality of life [178, 181]. 

The positive association between CD and DCM may be explained by nutritional deficiencies (iron, carnitine), but also both conditions might be mediated through inflammation and autoimmune mechanisms. CD causes abnormalities of intestinal permeability, leading to an increased systemic absorption of various luminal antigens and infectious agents, which may cause myocardial damage through immune-mediated mechanisms. Myocardial injury can also be attributed to immune response cross-reactivity against antigens present in the small intestine and the myocardium [180, 182, 183].

A possible association between CD and pericarditis has been described, but only limited to a small number of case-reports with good response with GFD [184–186]. However, at present, this relationship has not been clearly demonstrated [187]. 

#### 3.6.2. Psoriasis

Psoriasis is a chronic and relapsing inflammatory disorder of the skin characterised by scaling, erythema, and less commonly postulation and was reported to be associated with systemic comorbidities [135]. 

Patients with psoriasis were found to have significantly higher rates of CD than the general population, with a prevalence reaching up to 4.34% in this group of patients [188]. It has also been reported that in some cases a GFD may improve the skin lesions with no additional pharmacological treatment, particularly in patients with CD-associated antibodies, and these same patients may experience flare-ups with the reintroduction of this protein in the diet [189, 190]. 

A recent nationwide cohort study has confirmed CD as a risk factor for developing psoriasis with a HR of 1.72 (95% CI, 1.54–1.92). But in this study, it is noteworthy that this association between CD and psoriasis was found to be independent of a temporal relationship, and this increased risk of developing psoriasis was seen both before and after CD diagnosis [191]. 

Several mechanisms might explain the positive association between both diseases, including increased and impaired intestinal permeability, vitamin D deficiency due to malabsorption in CD that predisposes to psoriasis and exposure to gliadin as a trigger factor for CD4 + T-cell response [135, 192]. 

#### 3.6.3. Sarcoidosis

Sarcoidosis is a chronic idiopathic granulomatous disease as a consequence of altered immune response to unidentified antigens. Sarcoidosis mainly involves the lungs, even though other organs may be affected [193].

Sarcoidosis has been associated with different autoimmune disorders and among them with CD, as both diseases seem to share some immunological and genetic disorders [194–197]. CD has been associated with an increased risk of sarcoidosis (HR 4.03; 95% CI, 2.32–7.00). Similarly, a prior sarcoidosis diagnosis is associated with an increased risk of CD (OR 3.58; 95% CI, 1.98–6.45) [198]. 

#### 3.6.4. Hematological Disorders

Idiopathic thrombocytopenic purpura (ITP) is an autoantibody-mediated condition characterized by an abnormally low number of platelets in the circulating blood [199].

The first report of an association between ITP and CD was made in 1982 [200]. Since then, other cases have been published linking these two diseases [201, 202], and it has been suggested that the common mechanism may be genetic through HLA system [200]. A recent cohort study also found that individuals with CD had an increased risk of ITP (HR 1.91; 95% CI, 1.19–3.11) [203]. 

#### 3.6.5. Thromboembolic Phenomena

CD is associated with hypercoagulability status. Thromboembolism [204], pregnancy loss [205], small bowel infarction [206], atrial fibrillation [207], Budd-Chiari syndrome [73], portal and splenic vein thrombosis [208], and cardiovascular disease [209, 210] have been described in celiac patients. Several mechanisms have been implicated in these thromboembolic disorders such as hyperhomocysteinemia in untreated CD, methylenetetrahydrofolate reductase variants, the high homology between factor XIII and tTG, protein C and S deficiencies due to vitamin K malabsorption, and high levels of thrombin-activatable fibrinolysis inhibitor [211–214]. Recently, it has been suggested that intestinal injury, endothelial dysfunction, platelet abnormalities, and enhanced apoptosis cause increased exposure of phospholipids or new epitopes, which are the origin of antiphospholipid, antiprothrombin, and antiphosphatidylserine/prothrombin autoantibodies. The levels and activities of these antibodies are increased in celiac patients and might also play a pathogenic role in the thrombophilia associated with this disease [75].

#### 3.6.6. Pancreatic Disease

It has been described that patients with CD have an increased risk of both acute and chronic pancreatitis [215–217], with an HR for gallstone-related acute pancreatitis of 1.59 (95% CI, 1.06–2.40), for nongallstone-related acute pancreatitis of 1.86 (95% CI, 1.52–2.26), for chronic pancreatitis of 3.33 (95% CI, 2.33–4.76), and for supplementation with pancreatic enzymes of 5.34 (95% CI, 2.99–9.53). The risk of any pancreatitis within 5 years of CD diagnosis was estimated in 2.76 (95% CI, 2.36–3.22) [217]. It is noteworthy that exocrine pancreatic insufficiency is associated with persisting diarrhea in adult celiac patients [218]. 

The mechanisms that may be involved in the relationship between pancreatic disease and CD including impaired secretion of pancreatitis stimulating hormones from the diseased small bowel, alterations in enteric endocrine cells, reduction in precursors for pancreatic enzyme synthesis, structural changes in the pancreas with atrophy of acinar cells and fibrosis of the gland resulting in impaired pancreatic exocrine function, papillary stenosis and shared immunologic traits in both diseases [215, 219]. By contrast, the relationship between autoimmune pancreatitis and CD has not been demonstrated, since there is only so far a case report of this association [220].

#### 3.6.7. Microscopic Colitis

Some case reports and series have shown an association between CD and microscopic colitis [221–224]. Up to 15% of patients with collagenous or lymphocytic colitis have CD [221], and conversely, microscopic colitis was found in 4% of celiac patients representing up to a 70-fold increased risk for individuals with CD compared with the general population [222, 224]. Therefore, CD should be excluded in all patients with microscopic colitis, particularly if diarrhea does not respond to conventional treatment. 

## 4. Enteropathy-Associated T-cell Lymphoma and Celiac Disease 

CD is also characterized by an increased mortality [225]. It is well known that this fact is mainly the result of the complications of CD itself, represented by refractory CD (RCD) and enteropathy-associated T-cell lymphoma (EATL) [226]. This is a rare complication (<1% of lymphomas) and has a poor prognosis.

RCD is a form of CD that does not respond histologically to at least 12 months of a strict GFD [227]. This can also evolve in patients who initially responded normally to a GFD and who are still maintaining a strict GFD. On the basis of IELs population, RCD is further classified into type 1 and type 2. RCD type 1 (RCD1) is characterized by persisting villous atrophy despite a strict GFD associated with increased but still phenotypically normal IELs. Conversely, a clonal expansion of abnormal IELs lacking surface CD3, CD8, and TCR markers, but expressing intracellular CD3, indicates RCD type 2 (RCD2), a condition that frequently evolves into EATL, the most serious complication of CD. Nevertheless, the RCD prevalence is low, being estimated to appear in around 1% of CD patients [228].

For RCD prognosis, there are no doubts that it is much worse than that of uncomplicated CD. The 5-year survival rate is reported to be between 80% and 96% in patients with RCD1, but it is only between 40% and 58% in patients with RCD2. Five-year survival dropped to between 8% and 20% in RCD2 patients who developed EATL [229, 230].

Although the incidence of EATL was reported to be rare in the general population (1 per 10^6^ person-years) [231], it was shown that it occurs in 60% to 80% of patients with RCD2 within 5 years [232]. The description of EATL arising in patients with RCD1 seems to be exceptional. Recent evidence suggests that non-EATLs, including intestinal B-cell and extraintestinal T-cell lymphomas, may rarely occur in celiac patients [233].

EATL manifests in adult patients with previously diagnosed CD, successfully treated until then with a strict GFD (secondary EATL), as an exacerbation of the classic symptoms of CD, such as abdominal pain, diarrhea, and unexplained weight loss. The concomitant presence of fever and night sweating, together with laboratory parameters indicative for hypoalbuminemia, anemia, and increased lactate dehydrogenase (LDH), should alert physicians to this complication. The median age at diagnosis of EATL is 60 years, with similar frequency between men and women. On the other hand, EATL may also arise in patients without a known history of CD and on a gluten-containing diet (primary EATL), and in these cases, the diagnosis is more difficult and delayed because of the low specificity of symptoms and the very low index of clinical suspicion. In the subjects with EATL identified before CD has been diagnosed, the link between CD and EATL may be suggested by the detection of CD-specific antibodies (either EMAs or anti-tTG), although the latter often disappears once the refractory state is fully developed [234].

Because EATL may be complicated by gastrointestinal perforation, obstruction, or hemorrhage, many EATLs are diagnosed at laparotomy. At gross examination, EATL appears as a massive tumor infiltration, which may be transparietal with ulcerations and induration of the intestinal wall. Up to 25% of cases have a multifocal presentation, and the proximal small bowel, particularly the jejunum, is a more common localization than the large bowel or rectum [235]. There are reports of an association between EATL and peripheral eosinophilia, mesenteric lymph node cavitation, or splenic atrophy, the latter of which may increase susceptibility to severe infections or sepsis [236]. Malnutrition is a common feature, especially when EATL has an insidious and chronic presentation or manifests after a long-standing RCD. Extraintestinal presentation of EATL is rare, and there is a lack of data on the precise characteristics of its cutaneous, neuromeningeal, or pulmonary manifestations. Systemic or B symptoms, such as fever of no evident cause, night sweats, and weight loss of more than 10% of body weight, should be taken as signs of clinical progression, although they occur in less than 30% of EATLs. A high level of clinical suspicion for an overt lymphoma should lead to an extensive workup, including abdominal imaging, endoscopy, and histologic examination of gut biopsies. Laparotomy with collection of full-thickness biopsy specimens may be necessary in some cases.

Poor adherence to a GFD, *HLA-DQ2* homozygosity, and late diagnosis of CD are recognized as risk factors for malignant evolution of CD. The suspicion of EATL should lead to an extensive diagnostic workup in which magnetic resonance enteroclysis, positron emission tomography scan, and histologic identification of lesions represent the best options. Treatment includes high-dose chemotherapy preceded by surgical resection and followed by autologous stem cell transplantation, although biologic therapies seem to be promising. Strict adherence to a GFD remains the only way to prevent EATL. 

## Figures and Tables

**Table 1:** Celiac disease and associated autoimmune diseases.

*Liver diseases *	*Rheumatological/connective tissue diseases *
(i) Primary biliary cirrhosis	(i) Rheumatoid arthritis
(ii) Autoimmune hepatitis	(ii) Juvenile rheumatoid arthritis/Juvenile idiopathic arthritis
(iii) Primary sclerosing hepatitis	(iii) Sjogren's syndrome
*Endocrine diseases *	(iv) Systemic lupus erythematosus
(i) Diabetes mellitus	*Cardiological diseases *
(ii) Autoimmune thyroid disease	(i) Dilated cardiomyopathy
(iii) Addison's disease	(ii) Autoimmune pericarditis
*Dermatological diseases *	*Others *
(i) Dermatitis herpetiformis	(i) Psoriasis
(ii) Alopecia areata	(ii) Sarcoidosis
(iii) Vitiligo	(iii) Immune thrombocytopenic purpura
(iv) Dermatomyositis	(iv) Pancreatitis
*Neurological diseases *	(v) Microscopic colitis
(i) Gluten ataxia	(vi) Enteropathy-associated T-cell lymphoma
(ii) Peripheral neuropathies	
